# Psychological adaptation to generative AI in service work: knowledge elaboration and future orientation

**DOI:** 10.1186/s40359-026-05477-3

**Published:** 2026-09-15

**Authors:** Xiaokai Li, Yifan Ou, Jihan Wang

**Affiliations:** 1 School of Management and Economics, North China University of Water Resources and Electric Power, Zhengzhou, 450046 China; 2 Postdoctoral Research Station, Luohe Central Hospital, Luohe, 462000 China; 3 Manchester China Institute (MCI), University of Manchester, Manchester, M13 9PL UK; 4 Department of Communication Science, University of Antwerp, Antwerp, 2000 Belgium; 5 School of Business, The University of Sydney, Sydney, NSW 2006 Australia

**Keywords:** Generative AI acceptance, Service performance, Knowledge elaboration, Future orientation, Sociotechnical systems theory, Human–AI interaction and collaboration

## Abstract

**Background:**

Generative AI (GenAI) is increasingly reshaping service work and creating new demands for employees’ cognitive and psychological adaptation to human–AI interaction and collaboration. Although prior research has examined the organizational consequences of GenAI, limited attention has been paid to how employees’ acceptance of GenAI is associated with service performance through cognitive adaptation and how this association varies across employees. Drawing on sociotechnical systems theory, this study examined whether knowledge elaboration serves as a cognitive adaptation mechanism linking GenAI acceptance to employee service performance, and whether future orientation strengthens this process.

**Methods:**

This study employed a two-wave, multi-source survey design. Data were collected from 890 supervisor–employee dyads in the service sector. Employees reported their GenAI acceptance, future orientation, and demographic information at Time 1, while employees reported knowledge elaboration and supervisors evaluated service performance at Time 2. The hypothesized mediation and moderated mediation models were tested using regression analysis and bootstrapping procedures.

**Results:**

GenAI acceptance was positively associated with knowledge elaboration, which in turn was positively related to employee service performance. The estimated indirect association between GenAI acceptance and service performance through knowledge elaboration was significant, while the direct association remained significant. In addition, future orientation moderated the relationship between GenAI acceptance and knowledge elaboration, such that this relationship was stronger among employees with higher future orientation. The significant index of moderated mediation indicated that the estimated indirect association between GenAI acceptance and service performance through knowledge elaboration was stronger at higher levels of future orientation.

**Conclusion:**

The findings suggest that the association between GenAI acceptance and service performance involves employees’ cognitive engagement with AI-generated knowledge and is stronger among employees with higher future orientation. This study advances understanding of psychological adaptation in AI-integrated work settings by highlighting knowledge elaboration as a key cognitive mechanism and future orientation as an important psychological boundary condition.

**Supplementary Information:**

The online version contains supplementary material available at https://doi.org/10.1186/s40359-026-05477-3.

## Introduction

Generative AI (GenAI) broadly refers to a class of AI systems capable of producing novel content—such as text, images, videos, and other media—in response to user prompts [1, 2]. Owing to its user-friendly interfaces, GenAI enables a wider range of individuals to engage in AI-supported tasks and problem solving with relative ease [3, 4]. As a result, GenAI is increasingly recognized as a practical means of improving productivity and enhancing work outcomes across diverse organizational contexts [5]. At the same time, the diffusion of GenAI introduces new cognitive and psychological adaptation demands for employees working in AI-enabled environments.

These adaptation demands are particularly salient in service firms, where value creation depends heavily on employees’ real-time problem solving, adaptive use of knowledge, and interactions with customers [3, 5]. In such settings, rapid and unpredictable technological change can be especially consequential. Employees often experience these changes as shifting job content, escalating skill requirements, and new human–AI collaboration demands, which challenge the sufficiency of existing knowledge, increase ambiguity, and require sustained cognitive and psychological adaptation [6]. By automating and augmenting tasks traditionally reliant on human judgment and creativity, GenAI is reshaping knowledge-intensive work and altering how knowledge is created, shared, and applied within organizations [1, 6]. These transformations are likely to have important implications for employee outcomes, particularly service performance. Given that employee service performance is vital to sustaining competitive advantage and long-term success in service industries [5], understanding how employees’ acceptance of GenAI relates to service performance is of clear theoretical and practical importance.

Despite the growing relevance of GenAI in organizational life, workplace research on employees’ adaptive responses to GenAI remains limited, particularly regarding whether knowledge elaboration helps explain the association between psychological acceptance of GenAI and service performance. Prior studies have linked GenAI to outcomes such as employee creativity [2], work productivity and quality [7], service innovation [8], perceived value [4], and employee expediency [9]. However, the psychological process through which employees’ acceptance of GenAI is associated with service performance remains insufficiently understood. This omission is important because, in service settings, employees’ ability to respond effectively to customer needs directly shapes organizational success.

A further gap concerns the mechanisms through which GenAI-related psychological responses are linked to employee outcomes. GenAI does not merely provide information; through interactive and conversational engagement, it can support employee learning by responding to user queries, offering coaching, providing contextual examples, and delivering in-depth explanations [1, 10]. However, the availability of these capabilities alone does not explain variation in employee service performance. Employees’ evaluations of GenAI as useful, easy to use, and manageable may shape the extent to which they cognitively engage with AI-generated knowledge. In this regard, knowledge elaboration may represent a cognitive adaptation mechanism through which employees make sense of, integrate, and apply AI-generated knowledge in service work. Knowledge elaboration refers to the extent to which individuals actively reflect on, interpret, integrate, and apply newly acquired knowledge in their work [11, 12]. It reflects a deeper cognitive process through which task-relevant information is exchanged, discussed, and transformed into actionable knowledge [12, 13]. Yet existing research rarely examines whether GenAI acceptance is linked to employee service performance through such knowledge-processing activities.

In addition, prior research has paid insufficient attention to the conditions under which the associations involving GenAI acceptance vary across employees. Specifically, the association between GenAI acceptance and knowledge elaboration—and the corresponding indirect association with service performance—may depend on individual psychological characteristics. One such characteristic is future orientation, defined as the extent to which individuals habitually focus on the future, think about long-term goals, and consider the future consequences of their current actions [14, 15]. As such, future orientation can be viewed as an adaptive psychological capacity that helps employees interpret technological disruption in terms of future learning and development opportunities. Accordingly, future orientation may shape the extent to which GenAI acceptance fosters knowledge elaboration and, in turn, service performance.

To address this issue, we examine knowledge elaboration as a mediating mechanism linking GenAI acceptance to employee service performance and future orientation as a boundary condition shaping this relationship. Accordingly, we ask: How is GenAI acceptance associated with employees’ service performance through knowledge elaboration as a cognitive adaptation process, and how does this relationship vary as a function of future orientation?

Taken together, these gaps point to three important yet insufficiently understood questions concerning employees’ adaptation to GenAI-enabled service work. Accordingly, this study addresses the following research questions:

RQ1: How is GenAI acceptance associated with employee service performance?

RQ2: Does knowledge elaboration function as a cognitive adaptation mechanism linking GenAI acceptance to employee service performance?

RQ3: How does future orientation condition the relationship between GenAI acceptance and knowledge elaboration and, consequently, the indirect relationship between GenAI acceptance and employee service performance?

To address these questions, we draw primarily on sociotechnical systems (STS) theory and complement it with the knowledge-based view to develop a moderated mediation model. STS theory proposes that work outcomes arise from the joint functioning and alignment of technical and social subsystems rather than from technology alone [16–18]. Accordingly, we conceptualize employees’ adaptation to GenAI-enabled service work as a sociotechnical and psychological process rather than a simple technological input–performance output relationship. In this model, GenAI represents the technical resource, while GenAI acceptance reflects employees’ psychological alignment with this resource in terms of its perceived usefulness, ease of use, and manageability. Knowledge elaboration captures the cognitive adaptation process through which employees interpret, integrate, and apply AI-generated knowledge in their service work, whereas future orientation represents an individual psychological capacity that strengthens this adaptation process. Employee service performance, in turn, constitutes a behavioral outcome of the effective alignment among technological resources, psychological acceptance, cognitive engagement, and individual adaptive capacity. Complementing this STS logic, the knowledge-based view suggests that technological resources generate value only when employees can interpret, integrate, and transform available knowledge into effective work actions [19]. Together, these perspectives enable us to explain how GenAI acceptance is associated with employee service performance through knowledge elaboration and why this process is stronger among employees with higher future orientation.

We test this model using a two-wave, multi-source survey of 890 supervisor–employee dyads in the service sector. By temporally separating the measurement of predictor and mediator variables and collecting service performance ratings from supervisors, this design helps reduce common method concerns and strengthens the basis for examining the proposed relationships [20]. This study makes three main contributions. First, it shows that employees’ adaptive responses to GenAI in service contexts are best understood as a sociotechnical and psychological process rather than as a direct technological effect [18]. Second, it extends the knowledge-based view by identifying knowledge elaboration as a cognitive adaptation mechanism through which GenAI acceptance is associated with employee service performance [1, 10, 19]. Third, it identifies future orientation as an adaptive psychological capacity that conditions the strength of the indirect association between GenAI acceptance and service performance through knowledge elaboration [14, 15]. These contributions also provide practical insight into how service organizations can derive greater value from GenAI by aligning technological implementation with employees’ acceptance, learning, knowledge use, and customer-facing work demands [21, 22].

## Theory and hypotheses

### Sociotechnical systems theory and the proposed model

STS theory posits that organizational outcomes arise from the joint functioning of technical and social subsystems rather than from either subsystem in isolation [16–18]. The technical subsystem comprises technologies, processes, and related infrastructure, whereas the social subsystem encompasses employees, teams, relationships, and the psychological and behavioral processes through which work is performed. Accordingly, the value of technology depends not only on its technical functionality, but also on how effectively it is integrated with employees’ cognitive and behavioral responses.

This perspective is particularly relevant to GenAI-enabled service work. GenAI constitutes a technical resource that provides suggestions, explanations, contextual examples, and problem-solving support [1, 10]. However, the availability of these resources alone does not necessarily correspond to higher service performance. In our model, GenAI acceptance reflects employees’ psychological alignment with the technical resource, whereas knowledge elaboration represents the cognitive process through which AI-generated knowledge is interpreted, evaluated, integrated, and incorporated into service actions. Employee service performance is not treated merely as an organizational performance indicator. Rather, it is conceptualized as the behavioral manifestation of employees’ psychological acceptance of GenAI and cognitive engagement with AI-generated knowledge in customer-facing work. This interpretation is consistent with research linking employee–AI interaction to frontline employees’ service behaviors and performance [23]. Future orientation, in turn, functions as an individual adaptive capacity that conditions the strength of the association between psychological acceptance and cognitive engagement.

This conceptualization is also consistent with research showing that workplace AI outcomes reflect the interplay among technological capabilities, employee responses, and implementation contexts [18, 24]. Complementing this STS perspective, the knowledge-based view explains why technological resources become relevant to work outcomes only when employees interpret, integrate, and apply the knowledge they provide [19].

### GenAI acceptance and knowledge elaboration

GenAI acceptance is likely to be positively related to knowledge elaboration because employees who perceive GenAI as useful, easy to use, and manageable should be more willing to engage cognitively with its outputs. GenAI can respond to user queries, provide contextual examples and explanations, and generate alternative solutions in real time [1, 10]. However, employees are unlikely to elaborate on AI-generated knowledge unless they regard GenAI as a helpful and usable resource and invest cognitive effort in processing its outputs.

Knowledge elaboration refers to the extent to which individuals actively reflect on, interpret, integrate, and apply newly acquired knowledge in their work [11, 12]. In the present context, it represents a cognitive adaptation process through which employees clarify AI-generated information, evaluate its relevance, connect it with existing knowledge, and incorporate it into service practices.

At the construct level, GenAI acceptance reflects three closely related evaluative judgments. Perceived usefulness signals that AI-generated knowledge is relevant to employees’ work goals and has instrumental value, thereby motivating them to examine and apply it [25]. Perceived ease of use reduces the effort required to interact with GenAI, making sustained engagement with its outputs more feasible [25, 26]. Perceived manageability enhances employees’ sense of control over GenAI use and reduces uncertainty, a logic consistent with research on perceived external control in technology use [26]. Together, these judgments should increase employees’ willingness to engage deeply with AI-generated knowledge. Such engagement involves reflecting on, evaluating, verifying, refining, and integrating new information into existing knowledge structures [11–13].

From an STS perspective, GenAI acceptance therefore reflects employees’ psychological alignment with the technical resource, whereas knowledge elaboration represents the cognitive process through which AI-generated knowledge becomes integrated into service work [18, 27]. Accordingly, we propose:

#### Hypothesis 1

(H1). GenAI acceptance is positively related to knowledge elaboration.

### Knowledge elaboration and employee service performance

Employee service performance depends heavily on employees’ ability to understand customer needs, respond appropriately in real time, and apply relevant knowledge effectively in customer interactions [21, 22]. In GenAI-enabled service settings, the mere availability of information is insufficient to ensure high performance because employees must respond to customer needs through context-sensitive judgment and action. Rather, they must be able to make sense of the information they receive and convert it into effective service actions, as prior research suggests that the value of digital information for customer service performance depends on its translation into localized capabilities and responsive service behaviors [11–13, 28]. Knowledge elaboration is particularly important in this regard because it reflects a cognitive adaptation process through which employees move beyond passive receipt of information and translate knowledge into contextually appropriate service actions.

Employees who engage in greater knowledge elaboration are more likely to interpret service-related information accurately, integrate new insights with existing expertise, and apply them flexibly to customer needs. Prior research suggests that deeper information and knowledge processing is associated with more effective integration of available knowledge into task performance and decision making [11, 13]. In addition, knowledge elaboration has been associated with more effective use of received knowledge and more favorable work-related outcomes [12]. Employees who engage in greater knowledge elaboration should therefore be better equipped to tailor their responses, address service problems, and provide context-sensitive service. Within the STS framework, knowledge elaboration represents the human-side integration process through which AI-generated outputs are incorporated into customer-facing service actions. Employee service performance, in turn, represents the behavioral outcome associated with this sociotechnical integration process [23]. Therefore, we propose:

#### Hypothesis 2

(H2). Knowledge elaboration is positively related to employee service performance.

### The mediating role of knowledge elaboration

Taken together, the preceding arguments suggest that knowledge elaboration serves as a cognitive adaptation pathway linking GenAI acceptance to employee service performance. STS theory holds that technological resources generate value only when they are effectively integrated with human cognition and work practices [16–18]. In the present context, GenAI provides employees with informational resources, but these resources are unlikely to enhance service performance automatically. Employees who accept GenAI as useful, easy to use, and manageable should be more willing to engage with its outputs and integrate AI-generated knowledge into their work.

Knowledge elaboration involves reflecting on AI-generated information, evaluating its relevance, combining it with existing knowledge, and incorporating it into context-sensitive service actions. This reasoning is consistent with the knowledge-based view, which emphasizes that the value of available knowledge depends on its effective interpretation, integration, and application [19]. It is also consistent with evidence that digital information contributes to customer service performance when it is converted into localized capabilities and responsive service processes [28]. Thus, GenAI acceptance reflects employees’ psychological alignment with the technical resource, knowledge elaboration facilitates its cognitive integration into service practices, and service performance represents the behavioral outcome associated with this sociotechnical process. Accordingly, we propose:

#### Hypothesis 3

(H3). Knowledge elaboration mediates the relationship between GenAI acceptance and employee service performance.

### The moderating role of future orientation

Although GenAI acceptance is expected to be positively related to knowledge elaboration, this relationship may vary across employees. Future orientation refers to the extent to which individuals habitually focus on long-term goals and consider the future consequences of their current actions [14, 15]. Future-oriented individuals tend to invest greater effort in learning and proactive behaviors whose benefits may emerge over time [29]. In GenAI-enabled service work, future orientation can therefore be understood as an individual adaptive capacity that shapes how employees respond to an evolving technical resource. Importantly, future orientation is expected not merely to promote knowledge elaboration directly, but to condition the extent to which employees’ psychological acceptance of GenAI develops into cognitive engagement with its outputs.

Specifically, employees with higher future orientation are more likely to view GenAI as a resource for long-term learning, skill development, and career adaptation rather than merely as a tool for immediate task completion. Consequently, when they accept GenAI as useful, easy to use, and manageable, they should be more willing to invest cognitive effort in interpreting, evaluating, and integrating its outputs. Recent qualitative research similarly indicates that employees proactively adapt their work practices in response to GenAI while considering its implications for skill development and career prospects [30]. By contrast, employees with lower future orientation may interpret GenAI acceptance primarily in terms of immediate task utility and invest less cognitive effort in elaborating on its knowledge outputs. Thus, among employees with comparable levels of GenAI acceptance, those with higher future orientation should engage more deeply with AI-generated knowledge than those with lower future orientation. From an STS perspective, future orientation thus strengthens the extent to which employees’ psychological alignment with the technical resource develops into cognitive integration. Therefore, we propose:

#### Hypothesis 4

(H4). Future orientation moderates the positive relationship between GenAI acceptance and knowledge elaboration, such that the relationship is stronger for employees with higher future orientation.

Extending this STS logic, future orientation should strengthen the indirect relationship between GenAI acceptance and employee service performance by increasing the extent to which employees convert psychological acceptance of the technical resource into knowledge elaboration. Thus, the proposed sociotechnical alignment process should be stronger among employees with higher future orientation. Accordingly, we propose:

#### Hypothesis 5

(H5). Future orientation positively moderates the indirect effect of GenAI acceptance on employee service performance through knowledge elaboration, such that the indirect effect is stronger for employees with higher future orientation.

**Fig. 1:**
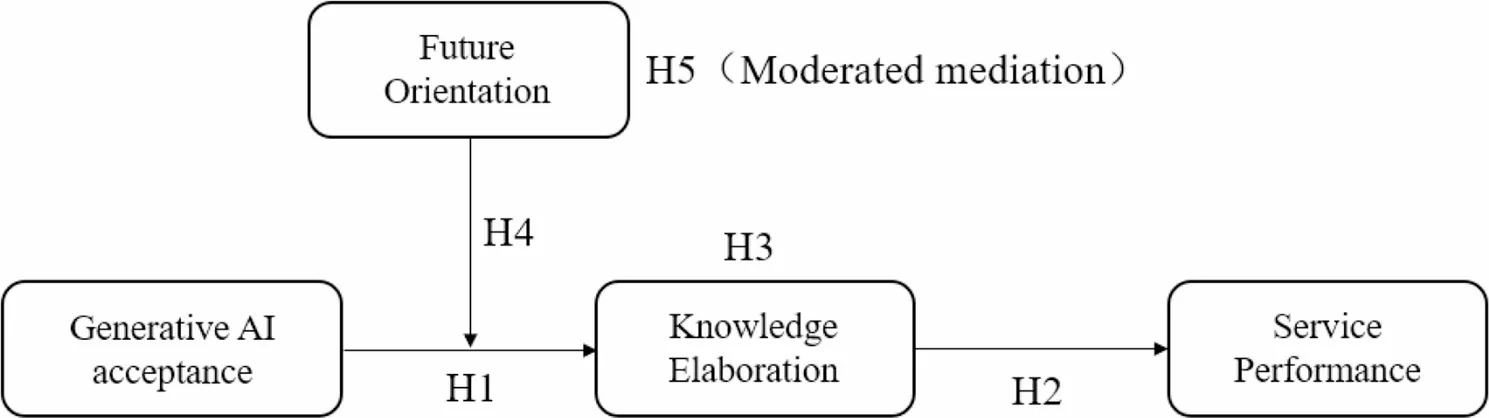
The proposed research model

Overall, Fig. 1 presents the proposed research model.

## Method

### Participants and procedure

Because this study focuses on workplace GenAI use, we collected data from employees in Chinese service-sector companies that had implemented GenAI tools. All sampled employees were using GenAI in their work at the time of data collection. We recruited supervisor–employee dyads from a variety of service-sector firms through professional and personal networks. Before data collection, we contacted supervisors in the participating firms and explained the purpose and procedures of the study. These supervisors were then asked to invite their immediate subordinates to participate.

To reduce potential common method bias, we adopted a two-wave, multi-source design and several procedural remedies [20, 31]. First, employees reported GenAI acceptance, future orientation, and demographic information at Time 1 and knowledge elaboration three months later at Time 2, providing temporal separation between the predictor and mediator measures. Second, service performance was rated by supervisors rather than by employees. Third, participants were assured that their responses would remain confidential, would be used only for research purposes, and would not be disclosed to their supervisors or organizations. Unique identification codes were used solely to match employee responses across waves with supervisor ratings.

Of the initial respondents, 54 did not participate at Time 2, 36 completed the Time 2 employee survey but could not be matched with a corresponding supervisor rating, and 30 were excluded because their Time 2 employee or supervisor responses were incomplete. These exclusion categories were mutually exclusive. The final sample consisted of 890 supervisor–employee dyads, representing a retention rate of 88.1%.

Following recommendations for assessing attrition in longitudinal research [32], we compared retained and excluded participants using the Time 1 variables available for both groups. Independent-samples t tests and chi-square tests revealed no significant differences in gender, age, education, tenure, GenAI acceptance, or future orientation (all ps > 0.05). These results suggest that systematic attrition was unlikely to have substantially biased the final sample. Table 1 presents the demographic characteristics of the sample.

**Table 1:** Demographic information

Feature	Category	%	*N*	Feature	Category	%	*N*
Gender	Male	53.9	480	Age	20–30 years	33.7	300
	Female	46.1	410		30–40 years	51.7	460
Tenure	1 year or below	7.5	67		40–50 years	12.4	110
	1–2 years	15.4	137		50–60 years	2.2	20
	3–4 years	21.3	190	Education	Junior high school and below	0.7	6
	5–7 years	17.3	154		High school	4.3	38
	More than 7 years	38.4	342		Associate degree	13.5	120
Firm size	Below 100 employees	21.1	188		Bachelor’s degree	54.0	481
	100–300 employees	22.5	200		Graduate degree	27.5	245
	300–500 employees	17.9	159	Industry	Hotel and catering	44.9	400
	500–1,000 employees	7.2	64		Tourism services	3.6	32
	More than 1,000 employees	31.3	279		Retail	7.8	69
Firm age	5 years or below	10.8	96		Information technology	4.2	37
	6–10 years	16.1	143		Financial services	7.5	67
	11–15 years	15.4	137		Logistics and transportation	7.5	67
	16–20 years	13.6	121		Education and training	5.8	52
	More than 20 years	44.2	393		Others	18.7	166

### Measures

All focal constructs were measured using established scales and assessed on a seven-point Likert scale ranging from 1 (strongly disagree) to 7 (strongly agree). We followed Brislin’s [33] translation and back-translation procedure to develop the Chinese questionnaire. GenAI acceptance and future orientation were reported by employees at Time 1, whereas knowledge elaboration was reported by employees and service performance was rated by supervisors at Time 2.

The GenAI acceptance measure was adapted from Davis [25] and contextualized for GenAI use based on Singh et al. [34] and Khan et al. [35]. Although all respondents had workplace experience with GenAI, the modal wording “would” was retained to preserve the evaluative framing of the adapted items. Accordingly, the measure captures experience-based evaluations of GenAI’s usefulness, ease of use, and manageability rather than the frequency or intensity of actual use. The knowledge elaboration items were adapted from Ling et al. [12] by replacing the original human knowledge source with GenAI. Future orientation was measured using Shipp et al.‘s [14] scale, whereas service performance was assessed using items from Liao and Chuang [21]. The complete measurement items, sources, standardized factor loadings, and reliability and validity statistics are presented in Supplementary Table S1.

At Time 1, employees also reported the control variables used in the analyses. Drawing on prior service-performance research [21, 22], we controlled for employee education and tenure, as well as firm size and firm age.

### Analysis strategy

Descriptive statistics, scale reliability, and correlation analyses were conducted using SPSS 27.0, while the heterotrait–monotrait ratio (HTMT) was calculated using Mplus 8.0. H1, H2, and H4 were tested using OLS regression analyses, which are appropriate for examining relationships among observed composite variables [36]. H3 and H5 were tested using Hayes’ [37] PROCESS macro for SPSS, with Model 4 for mediation and Model 7 for moderated mediation. To statistically assess potential common method bias, we compared the hypothesized three-factor model for the employee-reported constructs with a single-factor model in which all GenAI acceptance, knowledge elaboration, and future orientation items loaded on one factor. Percentile bootstrap confidence intervals based on 5,000 resamples were used to assess the indirect effects and the index of moderated mediation. All reported regression coefficients are unstandardized coefficients (b). GenAI acceptance and future orientation were mean-centered before constructing the interaction term. Conditional effects were probed at the 16th, 50th, and 84th percentiles of future orientation, corresponding to FO values of 5, 6, and 7, respectively. These values represent relatively low, median, and relatively high observed levels rather than the conventional mean ± 1 SD values [37].

## Results

### Descriptive statistics

Table 2 presents the means, standard deviations, and correlations among the study variables. The zero-order correlations provide preliminary support for Hypotheses 1 and 2. Specifically, GenAI acceptance was positively correlated with knowledge elaboration (*r* = 0.483, *p* < 0.001), and knowledge elaboration was positively correlated with service performance (*r* = 0.467, *p* < 0.001).

**Table 2:** Means, standard deviations and correlations for the study variables

Variables	Mean	SD	1	2	3	4	5	6	7
1.Education	4.035	0.801							
2.Tenure	3.637	1.327	-0.002						
3.Firm age	3.643	1.443	-0.032	0.188***					
4.Firm size	3.052	1.548	0.058	0.184***	0.507***				
5.Service performance	5.769	1.088	-0.028	0.079*	0.021	0.053			
6.GenAI acceptance	5.648	1.250	0.008	0.045	0.028	0.057	0.483***		
7.Knowledge elaboration	5.069	1.308	0.019	-0.045	0.009	-0.001	0.467***	0.483***	
8.Future orientation	5.807	1.068	0.039	0.029	-0.001	0.034	0.556***	0.554***	0.457***

*N* = 890; ^*^p < 0.05, ^**^p < 0.01, ^***^p < 0.001

### Measurement model

Before hypothesis testing, we conducted confirmatory factor analyses using the diagonally weighted least squares estimator. As shown in Table 3, the hypothesized four-factor model provided the best fit, χ²(84) = 664.540, *p* < 0.001, CFI = 0.988, TLI = 0.985, RMSEA = 0.081, and SRMR = 0.027. The four-factor model outperformed all alternatives, supporting construct distinctiveness. As shown in Supplementary Table S1, the standardized factor loadings ranged from 0.856 to 0.972 and were all significant. Cronbach’s alpha values ranged from 0.926 to 0.932, composite reliability values ranged from 0.953 to 0.956, and AVE values ranged from 0.805 to 0.877. Thus, all factor loadings and reliability estimates exceeded 0.70, and all AVE values exceeded 0.50, supporting satisfactory internal consistency and convergent validity.

**Table 3:** Results of confirmatory factor analyses

Models	χ²	df	χ²/df	CFI	TLI	RMSEA	SRMR
Four-factor model: GAI; KE; FO; SP	664.540	84	7.911	0.988	0.985	0.081	0.027
Three-factor model: GAI; KE; FO + SP	2366.318	87	27.199	0.952	0.942	0.172	0.088
Two-factor model: GAI; KE + FO+SP	4436.873	89	49.853	0.908	0.892	0.234	0.146
Single-factor model: GAI + KE+FO + SP	5931.678	90	65.907	0.877	0.856	0.270	0.192

GAI = GenAI acceptance; KE = knowledge elaboration; FO = future orientation; SP = service performance; CFI = comparative fit index; TLI = Tucker–Lewis index; RMSEA = root mean square error of approximation; SRMR = standardized root mean square residual. All χ² values were significant at *p* < 0.001. *N* = 890

As additional evidence of discriminant validity, we assessed the four focal constructs using the heterotrait–monotrait ratio (HTMT), which compares correlations across constructs with correlations among indicators of the same construct [38]. As shown in Table 4, the HTMT values ranged from 0.492 to 0.600 and were all below the recommended threshold of 0.85. These results support the empirical distinctiveness of GenAI acceptance, knowledge elaboration, future orientation, and service performance [36].

A three-factor model for the employee-reported constructs, χ²(51) = 547.602, CFI = 0.987, TLI = 0.983, RMSEA = 0.105, and SRMR = 0.028, substantially outperformed a single-factor model, χ²(54) = 3857.120, CFI = 0.900, TLI = 0.878, RMSEA = 0.281, and SRMR = 0.191. Thus, a single common factor was unlikely to account for the covariance among the employee-reported measures. Taken together with the procedural remedies, these results suggest that common method variance was unlikely to seriously affect the main findings.

**Table 4:** Heterotrait–monotrait ratios among the focal constructs

Construct	1	2	3	4
1. GenAI acceptance	—			
2. Knowledge elaboration	0.519	—		
3. Future orientation	0.597	0.492	—	
4. Service performance	0.519	0.503	0.600	—

Values below the diagonal are heterotrait–monotrait ratios. All values are below the recommended threshold of 0.85

### Hypotheses testing

H1, H2, and H4 were tested using OLS regression (see Table 5). As reported in Table 5 (Model 1), the regression model predicting knowledge elaboration was statistically significant (F = 60.366, *p* < 0.001) and explained 28.6% of the variance in the dependent variable (adjusted R² = 0.286). GenAI acceptance was positively associated with knowledge elaboration (b = 0.352, *p* < 0.001), and future orientation was also positively associated with knowledge elaboration (b = 0.335, *p* < 0.001). These results provide support for H1.

**Table 5:** OLS regression results

	DV=Knowledge elaboration					DV=Service performance	
	Model 1: direct effect		Model 2 moderation effect			Model 3: direct effect	
	*b*	S.E		*b*	S.E	*b*	S.E
Intercepts	1.307***	0.307		5.181***	0.231	1.912***	0.239
**Controls**							
Education	0.014	0.047		0.024	0.046	-0.067	0.036
Tenure	-0.066*	0.029		-0.070*	0.029	0.056*	0.022
Firm age	0.027	0.030		0.024	0.030	-0.012	0.023
Firm size	-0.028	0.028		-0.029	0.028	0.021	0.022
**Direct effects**							
GenAI acceptance (GA)	0.352***	0.036		0.373***	0.036	0.150***	0.029
Future orientation (FO)	0.335***	0.042		0.371***	0.043	0.363***	0.033
Knowledge elaboration						0.187***	0.026
**Interaction effects**							
GA x FO				0.061**	0.019		
F value	60.366***			53.774***		82.161***	
R^2^	0.291			0.299		0.395	
Adjusted R^2^	0.286			0.294		0.390	

*N* = 890; ^*^p < 0.05, ^**^p < 0.01, ^***^p < 0.001. Entries are unstandardized regression coefficients

**Fig. 2:**
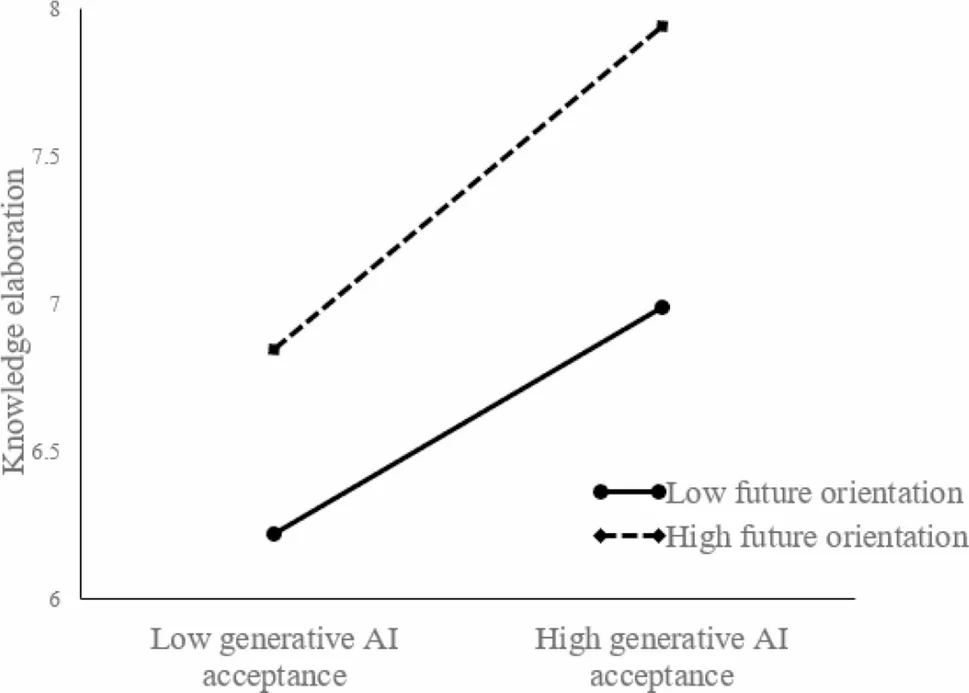
The moderating effect of future orientation

Model 2 introduced the interaction between mean-centered GenAI acceptance and mean-centered future orientation to predict knowledge elaboration. The model explained a substantial proportion of the variance in knowledge elaboration (adjusted R² = 0.294). The interaction term was significant (b = 0.061, *p* = 0.001), indicating that the positive association between GenAI acceptance and knowledge elaboration varied across levels of future orientation. Both mean-centered GenAI acceptance (b = 0.373, *p* < 0.001) and mean-centered future orientation (b = 0.371, *p* < 0.001) were positively associated with knowledge elaboration. Thus, H4 is supported. Figure 2 illustrates the moderating role of future orientation in the association between GenAI acceptance and knowledge elaboration. For visualization, low and high future orientation are plotted at one standard deviation below and above the mean, respectively.

As shown in Table 5 (Model 3), the regression model predicting service performance was statistically significant, explaining 39% of the variance in the dependent variable (adjusted R² = 0.390). Knowledge elaboration was positively associated with service performance (b = 0.187, *p* < 0.001). Future orientation also remained a significant positive predictor (b = 0.363, *p* < 0.001). Therefore, H2 is supported.

Using the PROCESS Model 4, we examined whether knowledge elaboration mediated the relationship between GenAI acceptance and service performance while controlling for education, tenure, firm age, and firm size. The model predicting knowledge elaboration was significant, explaining 23.9% of its variance (R² = 0.239). GenAI acceptance was positively associated with knowledge elaboration (b = 0.510, *p* < 0.001). The difference from the corresponding OLS estimate reflects the inclusion of future orientation as an additional predictor in Model 1 of Table 5. The model predicting service performance was also significant, explaining 31.3% of its variance (R² = 0.313). In the model predicting service performance, both GenAI acceptance (b = 0.284, *p* < 0.001) and knowledge elaboration (b = 0.261, *p* < 0.001) were positively associated with service performance. The estimated indirect effect through knowledge elaboration was 0.133, and its 95% bootstrap confidence interval excluded zero (BootLLCI = 0.099, BootULCI = 0.172). The direct association between GenAI acceptance and service performance also remained significant. This pattern was consistent with the proposed partial mediation model and supported H3. However, because knowledge elaboration and service performance were measured at the same wave, the indirect effect should not be interpreted as definitive evidence of temporal or causal mediation.

To test the proposed moderated mediation, we used PROCESS Model 7, in which the interaction between GenAI acceptance and future orientation predicted knowledge elaboration. The model explained 29.9% of the variance in knowledge elaboration (R² = 0.299), and the interaction term was significant (b = 0.061, *p* = 0.001), indicating that future orientation moderated the association between GenAI acceptance and knowledge elaboration. Conditional effects were examined at relatively low (16th percentile; FO = 5), median (50th percentile; FO = 6), and relatively high (84th percentile; FO = 7) levels of future orientation. The positive association between GenAI acceptance and knowledge elaboration increased from *b* = 0.324 (SE = 0.037, *p* < 0.001, 95% CI [0.252, 0.396]) at the 16th percentile to *b* = 0.385 (SE = 0.037, *p* < 0.001, 95% CI [0.313, 0.458]) at the 50th percentile and *b* = 0.446 (SE = 0.046, *p* < 0.001, 95% CI [0.356, 0.537]) at the 84th percentile. These results support H4.

For service performance, the model, which included knowledge elaboration and control variables, explained 31.3% of the variance (R² = 0.313). The direct association between GenAI acceptance and service performance remained significant (b = 0.284, *p* < 0.001), and knowledge elaboration was positively associated with service performance (b = 0.261, *p* < 0.001). These findings suggest that GenAI acceptance is linked to service performance both directly and indirectly through knowledge elaboration. The estimated indirect association between GenAI acceptance and service performance through knowledge elaboration varied across the examined levels of future orientation. The conditional indirect effect was 0.085 at the 16th percentile (FO = 5; BootLLCI = 0.056, BootULCI = 0.115), 0.101 at the 50th percentile (FO = 6; BootLLCI = 0.071, BootULCI = 0.134), and 0.116 at the 84th percentile (FO = 7; BootLLCI = 0.079, BootULCI = 0.160). None of the bootstrap confidence intervals included zero. The index of moderated mediation was 0.016, and its 95% bootstrap confidence interval excluded zero (BootLLCI = 0.003, BootULCI = 0.033). This significant index provided evidence for moderated mediation and supported H5.

## Discussion

### Discussion of key findings

This study yielded three key findings. First, GenAI acceptance was positively associated with knowledge elaboration, which in turn was positively related to supervisor-rated service performance. The significant indirect effect, together with the remaining direct association between GenAI acceptance and service performance, indicates that knowledge elaboration represents an important, but not exclusive, cognitive pathway through which employees’ acceptance of GenAI is associated with service performance. This finding complements prior research documenting positive productivity and performance outcomes associated with GenAI and employee–AI interaction [7, 23]. The present pattern further suggests that the association between GenAI acceptance and service performance involves employees’ active interpretation, evaluation, refinement, and integration of AI-generated knowledge [27].

Second, the positive association between GenAI acceptance and knowledge elaboration was stronger at higher levels of future orientation. Correspondingly, the estimated indirect association between GenAI acceptance and service performance through knowledge elaboration was also stronger at higher levels of future orientation. This finding indicates that employees with comparable levels of GenAI acceptance do not necessarily engage with its knowledge outputs to the same extent. Employees with stronger future orientation appear more likely to interpret GenAI as a resource for long-term learning, skill development, and career adaptation and therefore to invest more cognitive effort in its outputs. This interpretation is consistent with qualitative evidence that employees proactively reshape their work with GenAI while considering its implications for skill development and career prospects [30]. The present findings extend this research by identifying future orientation as a psychological boundary condition of the GenAI acceptance–knowledge elaboration relationship.

Third, the findings are consistent with the central STS argument that workplace outcomes associated with GenAI reflect the interplay between the technical resource and employees’ psychological and cognitive responses. GenAI acceptance reflects psychological alignment with the technical resource, knowledge elaboration facilitates the cognitive integration of AI-generated knowledge into service practices, and future orientation strengthens this adaptation process. The findings are therefore consistent with research emphasizing the context-dependent and multilevel nature of workplace AI outcomes [18, 24]. They extend this perspective by specifying an employee-level adaptation pathway through which GenAI acceptance is associated with service performance in customer-facing work.

From a psychological perspective, service performance in this study represents the behavioral manifestation of employees’ adaptation to GenAI-enabled work rather than merely a distal organizational outcome. Employees first form psychological evaluations of GenAI, cognitively engage with and elaborate on its outputs, and express this adaptation through context-sensitive service behaviors. This interpretation is consistent with research emphasizing that employees actively regulate cognitive effort, evaluate AI-generated outputs, and adapt their work practices when collaborating with GenAI [27, 30]. Accordingly, the findings advance psychological understanding of human–AI interaction by linking technology-related evaluations, cognitive knowledge processing, and future-oriented individual differences to observable work behavior.

### Theoretical contributions

This study makes three main theoretical contributions. First, it refines STS explanations of GenAI-enabled service work by specifying an employee-level adaptation process involving psychological acceptance, cognitive integration, and service performance. Rather than treating GenAI as a technical input with uniform performance consequences, our findings indicate that its value is associated with the alignment between the technical resource and employees’ psychological and cognitive responses. This finding extends STS research by showing how such alignment may unfold in customer-facing service work [18, 24].

Second, this study identifies knowledge elaboration as a theoretically relevant cognitive adaptation pathway consistent with the observed indirect association between GenAI acceptance and employee service performance. Prior research has documented the productivity, creativity, and service-related benefits associated with GenAI and employee–AI interaction [7, 23], but has provided less explanation of how employees transform AI-generated outputs into context-sensitive service actions. The significant indirect association is consistent with the argument that favorable evaluations of GenAI become relevant to service performance partly through employees’ reflection on, evaluation of, refinement of, and integration of its outputs. Because the direct association between GenAI acceptance and service performance remained significant, knowledge elaboration represents an important but nonexclusive explanatory pathway. This finding provides a process-oriented extension of the knowledge-based view [19, 27].

Third, this study identifies future orientation as an individual psychological boundary condition of GenAI-related cognitive adaptation. Employees with comparable levels of GenAI acceptance do not necessarily elaborate on AI-generated knowledge to the same extent. Those with stronger future orientation are more likely to interpret GenAI as a resource for future learning, skill development, and career adaptation and therefore engage more deeply with its outputs. This finding extends future orientation beyond its conventional role as a generalized motivational resource by showing how it conditions the relationship between technological acceptance and cognitive integration in GenAI-enabled work [14, 30].

### Practical implications

This study offers several practical implications for service organizations seeking to support employee adaptation to GenAI-enabled work. First, because GenAI acceptance was positively associated with knowledge elaboration, managers should help employees understand the usefulness, ease of use, and manageability of GenAI in specific service tasks. Training should use realistic customer-service scenarios to demonstrate how GenAI can support complaint handling, service recovery, and personalized recommendations. Rather than treating GenAI merely as a tool for automating isolated tasks, firms should embed it into customer-facing workflows in ways that support employees’ interpretation of information, problem solving, and service delivery [1, 10]. Such implementation should augment employee judgment and discretion rather than replace them, particularly because service performance depends heavily on employees’ real-time responsiveness to customer needs [21, 22].

Second, the estimated indirect association between GenAI acceptance and service performance was consistent with mediation through knowledge elaboration. Accordingly, organizations should focus not only on providing access to GenAI but also on helping employees process and apply AI-generated knowledge. Training should teach employees not merely how to obtain GenAI outputs, but how to verify their accuracy, refine their relevance, compare them with organizational policies and professional knowledge, and contextualize them for specific customer needs. Organizations can reinforce these capabilities by developing internal prompt libraries and case repositories and by introducing team debriefings or feedback routines in which employees discuss successful and unsuccessful AI-assisted service cases. These practices can help employees move beyond passive acceptance of AI-generated information and engage in deeper reflection, evaluation, and integration [11–13].

Third, because the positive association between GenAI acceptance and knowledge elaboration was stronger at higher levels of future orientation, a uniform implementation strategy may not be equally appropriate for all employees. Managers could invite highly future-oriented employees to serve as early adopters or peer mentors because they are more likely to view GenAI as a resource for long-term learning, skill development, and career adaptation [14, 30]. Employees with lower future orientation may benefit from more structured support, including coaching, guided practice, pilot programs, and staged implementation. Managers could also connect immediate GenAI-supported tasks with longer-term skill-development and career goals to encourage deeper engagement with AI-generated knowledge. More broadly, successful GenAI implementation should be treated as a sociotechnical change effort in which technological investment is accompanied by employee learning, managerial support, and workflow adaptation [18].

### Limitations and future research directions

Notwithstanding its contributions, this study has several limitations that suggest directions for future research. First, although the two-wave, multi-source design reduced common method concerns and temporally separated GenAI acceptance from knowledge elaboration, it does not establish the full temporal sequence implied by the mediation model. Knowledge elaboration and service performance were measured at the same wave, although from different sources. Consequently, the design cannot determine whether knowledge elaboration precedes service performance, whether stronger service performance promotes subsequent knowledge elaboration, or whether the relationship is reciprocal. Future research could use three-wave longitudinal designs, panel models, or experience-sampling methods to establish temporal ordering and examine how psychological acceptance, knowledge elaboration, and service performance evolve over time [39–41]. Accordingly, the significant indirect effect should be interpreted as being consistent with the proposed mediation model rather than as definitive evidence that knowledge elaboration temporally or causally transmits the effect of GenAI acceptance to service performance [42, 43].

Second, this study focused on GenAI acceptance rather than actual GenAI use. Acceptance captures employees’ evaluations of the usefulness, ease of use, and manageability of GenAI, whereas actual use involves behavioral dimensions such as frequency, intensity, duration, and task-specific application. Employees may hold favorable evaluations of GenAI without using it extensively, while frequent use may also result from organizational requirements rather than genuine psychological acceptance. Future research should therefore examine acceptance and actual use simultaneously and distinguish among different forms of GenAI use, such as automation and augmentation, because they may create different cognitive demands and rely on different knowledge-processing mechanisms [25, 44].

Third, the sample covered multiple service industries that may differ in customer-contact intensity, task characteristics, regulatory requirements, and opportunities for GenAI use. Moreover, a substantial proportion of respondents worked in the hotel and catering industry. Although the multi-industry sample provides contextual breadth, the findings may not generalize uniformly across all forms of service work because occupational and organizational contexts can shape employee behavior [45]. For example, knowledge elaboration may play a greater role in customized and knowledge-intensive services than in more standardized service settings. Future research could examine the model across service industries and test whether industry characteristics condition employee adaptation to GenAI.

Fourth, this study focused on employee service performance as the behavioral outcome of adaptation to GenAI-enabled work. However, GenAI may have broader psychological consequences that are not captured by service performance alone. Although GenAI can provide learning, information, and problem-solving resources, it may also increase cognitive demands, uncertainty, concerns about skill obsolescence, and dependence on technological systems. Future research could therefore examine psychological outcomes such as stress, well-being, resilience, thriving, and technology-related anxiety. It could also investigate whether knowledge elaboration and future orientation help employees cope with the psychological demands of human–AI collaboration or, under some conditions, intensify cognitive overload and strain [24, 46].

## Conclusion

Drawing on STS theory, this study examined how employees’ psychological and cognitive adaptation to GenAI-enabled service work is associated with service performance. The findings indicate that GenAI acceptance is linked to employee service performance both directly and indirectly through knowledge elaboration and that the estimated indirect association is stronger among employees with higher future orientation. These statistical patterns are consistent with the proposed moderated mediation model, although they should not be interpreted as definitive evidence of causal mediation. Overall, the findings suggest that GenAI-related service outcomes are associated not only with technological availability but also with employees’ psychological acceptance, cognitive engagement with AI-generated knowledge, and future-oriented capacity to adapt to technological change.

## Supplementary information

Below is the link to the electronic supplementary material.

Supplementary Material 1

## Data Availability

The data that support the findings of this study are available on request from the corresponding author. The data are not publicly available due to privacy/ethical considerations.
