# Supplementary material for: Psychological adaptation to generative AI in service work: knowledge elaboration and future orientation
Source: BMC Psychol. 2026 Sep 15;14:1603. doi: 10.1186/s40359-026-05477-3 (PMC13741578; doi:10.1186/s40359-026-05477-3)
Supplement: Supplementary file 1 [file 40359_2026_5477_MOESM1_ESM.docx]

Supplementary Material

Supplementary Table S1. Measurement items, sources, and standardized factor loadings

| Construct | Item | Item wording | Sources | Standardized factor loading | α | CR | AVE |
| --- | --- | --- | --- | --- | --- | --- | --- |
| GenAI acceptance | GAI1 | Generative AI adoption in my role would lead to a substantial improvement in organizational productivity. | Adapted from Davis [25], Singh et al. [34], and Khan et al. [35] | .899 | .932 | .954 | .805 |
|  | GAI2 | The utilization of generative AI tools would enable me to accomplish complex tasks more efficiently. |  | .924 |  |  |  |
|  | GAI3 | I would find it easy to instruct generative AI to perform specific tasks. |  | .856 |  |  |  |
|  | GAI4 | Generative AI would make it easier for me to perform my organizational responsibilities. |  | .920 |  |  |  |
|  | GAI5 | Becoming proficient in using generative AI would be a straightforward and manageable endeavor. |  | .885 |  |  |  |
| Knowledge elaboration | KE1 | I often consider the knowledge that GenAI shares with me and its possible applications in my work. | Adapted from Ling et al. [12] | .909 | .931 | .956 | .877 |
|  | KE2 | I seek clarification from GenAI about its responses and integrate the resulting information into my work. |  | .947 |  |  |  |
|  | KE3 | I integrate my own knowledge with knowledge provided by GenAI by considering the implications of the received information. |  | .953 |  |  |  |
| Future orientation | FO1 | I think about what my future has in store. | Shipp et al. [14]; Liang et al. [15] | .918 | .926 | .953 | .837 |
|  | FO2 | I think about times to come. |  | .911 |  |  |  |
|  | FO3 | I focus on my future. |  | .922 |  |  |  |
|  | FO4 | I imagine what tomorrow will bring for me. |  | .908 |  |  |  |
| Service performance | SP1 | This employee is friendly and helpful to customers. | Liao and Chuang [21]; Hur et al. [22] | .942 | .927 | .955 | .875 |
|  | SP2 | This employee points out services related to a customer's needs. |  | .972 |  |  |  |
|  | SP3 | This employee suggests items customers might like but did not think of. |  | .892 |  |  |  |

*Note.* All items were rated on a seven-point Likert scale ranging from 1 (strongly disagree) to 7 (strongly agree). GenAI acceptance, future orientation, and knowledge elaboration were reported by employees; service performance was rated by supervisors. All standardized factor loadings were significant at p < .001. CR = composite reliability; AVE = average variance extracted.
